# Supplementary material for: A Humanized Yeast Phenomic Model of Deoxycytidine Kinase to Predict Genetic Buffering of Nucleoside Analog Cytotoxicity
Source: Genes (Basel). 2019 Sep 30;10(10):770. doi: 10.3390/genes10100770 (PMC6826991; doi:10.3390/genes10100770)
Supplement: Supplementary file 1 [file genes-10-00770-s001.zip › Additional_Files/Additional_File7_GOTermHeatmaps.zip!/Additional_File7_GOTermHeatmaps/phosphatidylinositol_binding.pdf]

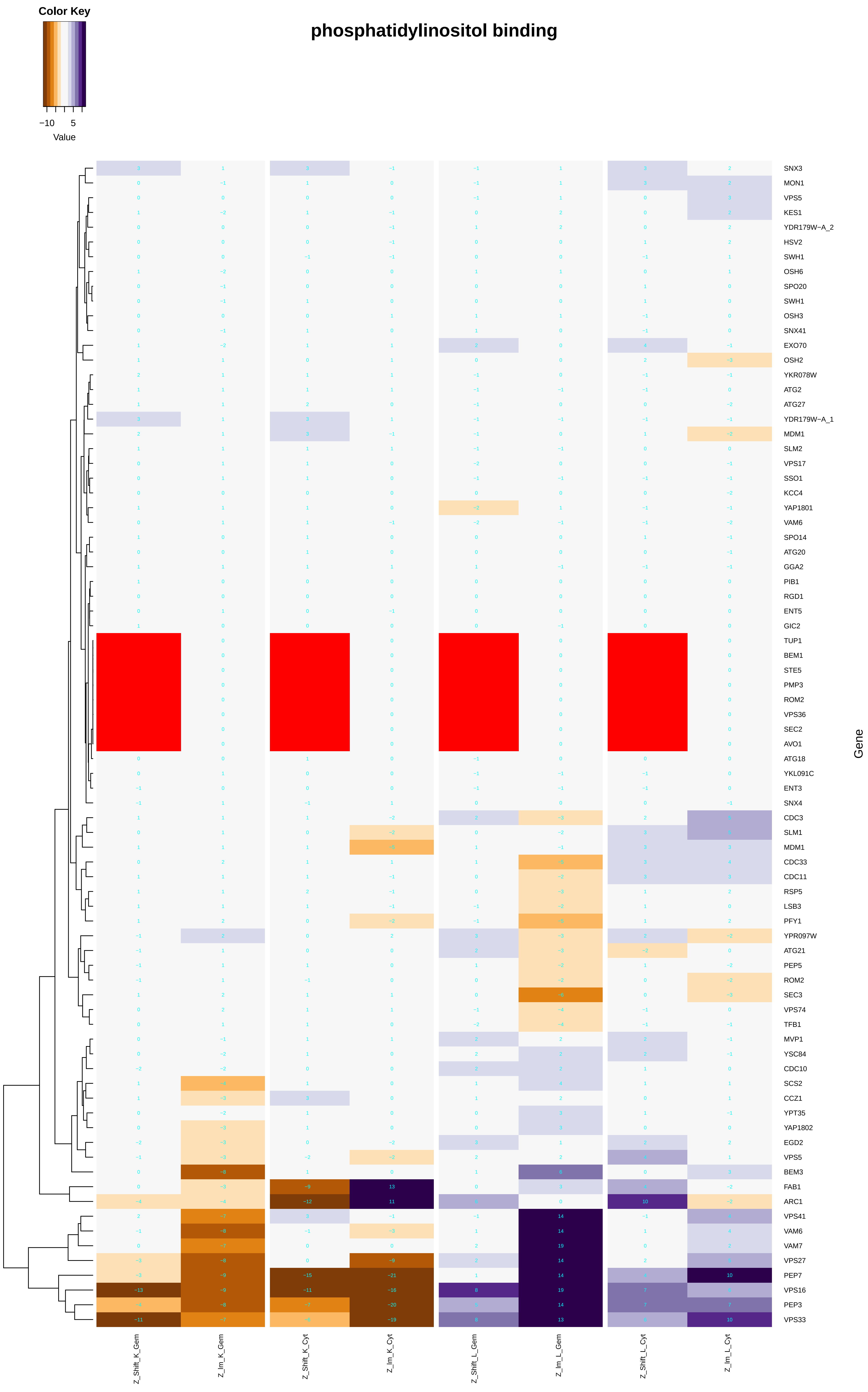
phosphatidylinositol binding
Color Key
−10
5
Value
Gene
SNX3
MON1
VPS5
KES1
YDR179W−A_2
HSV2
SWH1
OSH6
SPO20
SWH1
OSH3
SNX41
EXO70
OSH2
YKR078W
ATG2
ATG27
YDR179W−A_1
MDM1
SLM2
VPS17
SSO1
KCC4
YAP1801
VAM6
SPO14
ATG20
GGA2
PIB1
RGD1
ENT5
GIC2
TUP1
BEM1
STE5
PMP3
ROM2
VPS36
SEC2
AVO1
ATG18
YKL091C
ENT3
SNX4
CDC3
SLM1
MDM1
CDC33
CDC11
RSP5
LSB3
PFY1
YPR097W
ATG21
PEP5
ROM2
SEC3
VPS74
TFB1
MVP1
YSC84
CDC10
SCS2
CCZ1
YPT35
YAP1802
EGD2
VPS5
BEM3
FAB1
ARC1
VPS41
VAM6
VAM7
VPS27
PEP7
VPS16
PEP3
VPS33
Z_Shift_K_Gem
Z_Im_K_Gem
Z_Shift_K_Cyt
Z_Im_K_Cyt
Z_Shift_L_Gem
Z_Im_L_Gem
Z_Shift_L_Cyt
Z_Im_L_Cyt

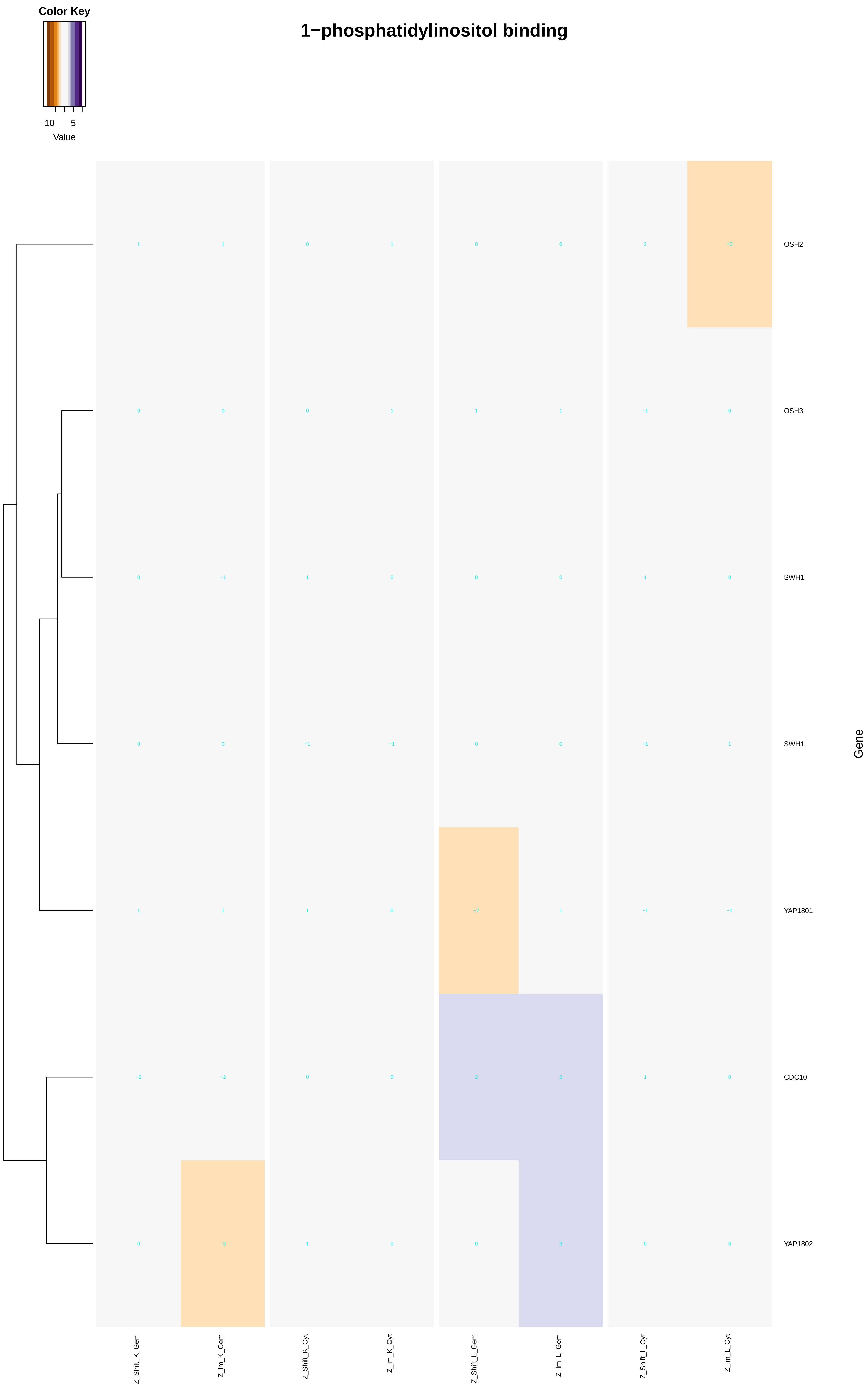

Color Key
−10
5
Value
1−phosphatidylinositol binding
OSH2
OSH3
SWH1
SWH1
YAP1801
CDC10
YAP1802
Gene
Z_Shift_K_Gem
Z_Im_K_Gem
Z_Shift_K_Cyt
Z_Im_K_Cyt
Z_Shift_L_Gem
Z_Im_L_Gem
Z_Shift_L_Cyt
Z_Im_L_Cyt

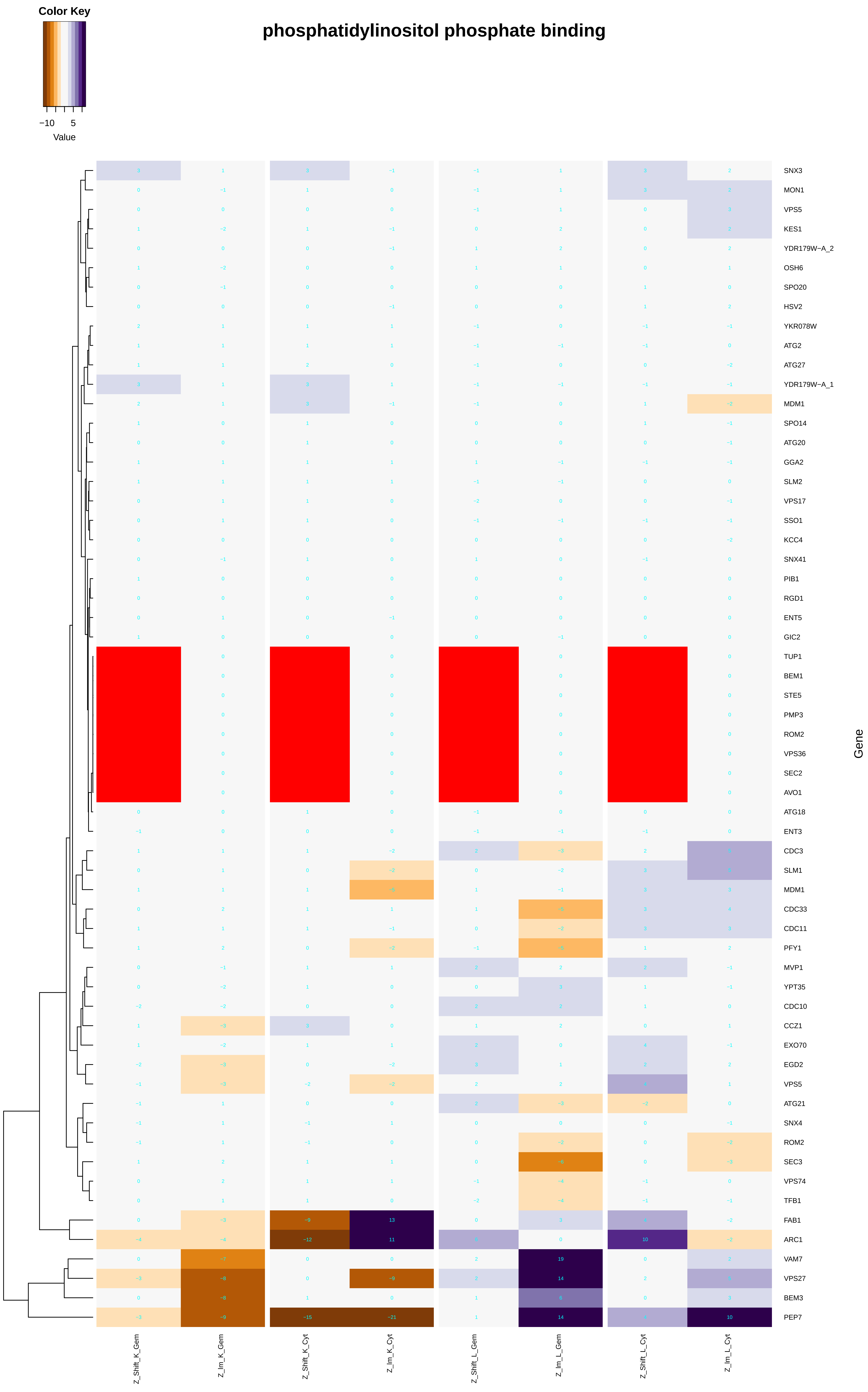
Color Key
−10
5
Value
phosphatidylinositol phosphate binding
SNX3
MON1
VPS5
KES1
YDR179W−A_2
OSH6
SPO20
HSV2
YKR078W
ATG2
ATG27
YDR179W−A_1
MDM1
SPO14
ATG20
GGA2
SLM2
VPS17
SSO1
KCC4
SNX41
PIB1
RGD1
ENT5
GIC2
TUP1
BEM1
STE5
PMP3
ROM2
VPS36
SEC2
AVO1
ATG18
ENT3
CDC3
SLM1
MDM1
CDC33
CDC11
PFY1
MVP1
YPT35
CDC10
CCZ1
EXO70
EGD2
VPS5
ATG21
SNX4
ROM2
SEC3
VPS74
TFB1
FAB1
ARC1
VAM7
VPS27
BEM3
PEP7
Gene
Z_Shift_K_Gem
Z_Im_K_Gem
Z_Shift_K_Cyt
Z_Im_K_Cyt
Z_Shift_L_Gem
Z_Im_L_Gem
Z_Shift_L_Cyt
Z_Im_L_Cyt

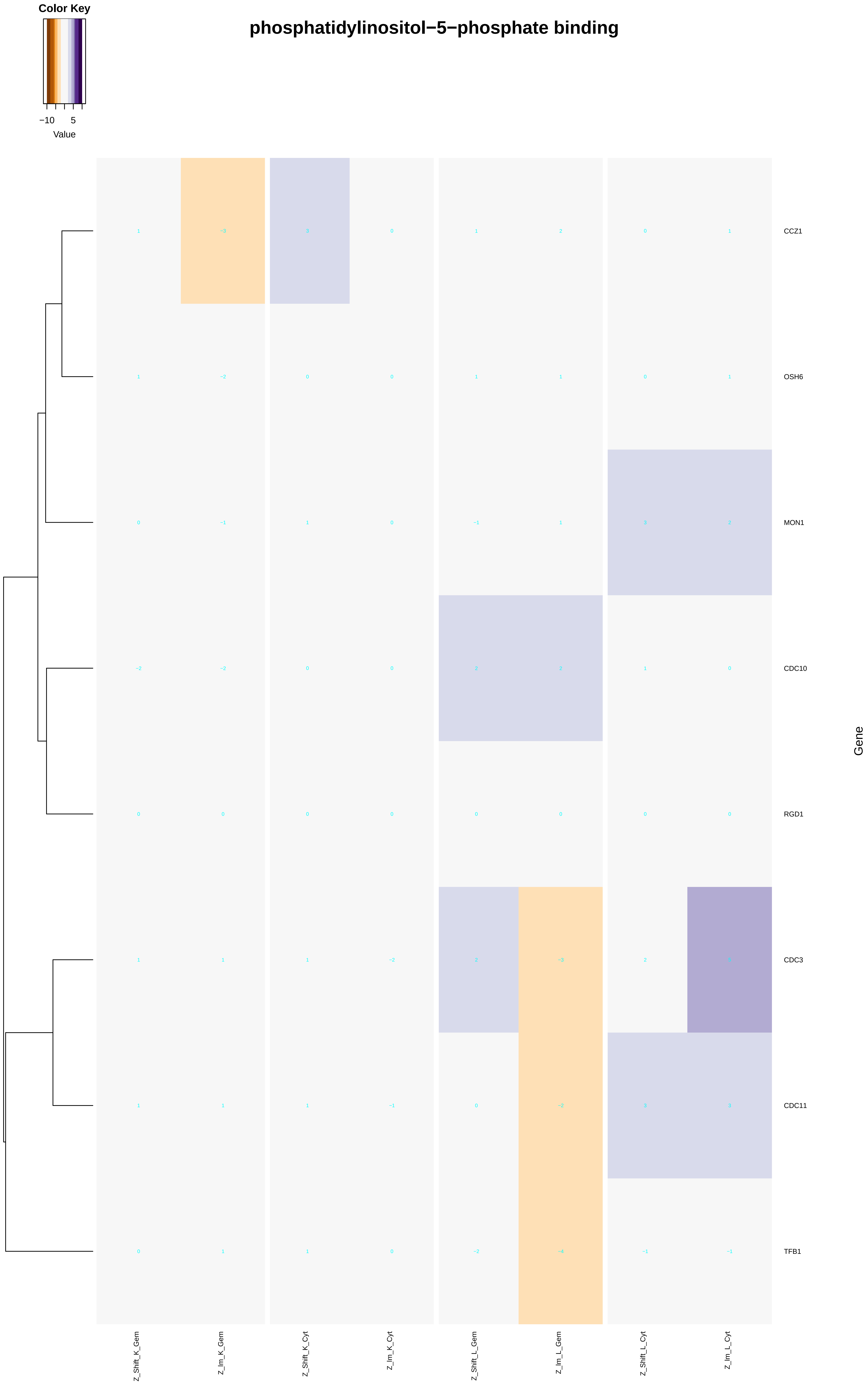
Color Key
−10
5
Value
phosphatidylinositol−5−phosphate binding
CCZ1
OSH6
MON1
CDC10
RGD1
CDC3
CDC11
TFB1
Gene
Z_Shift_K_Gem
Z_Im_K_Gem
Z_Shift_K_Cyt
Z_Im_K_Cyt
Z_Shift_L_Gem
Z_Im_L_Gem
Z_Shift_L_Cyt
Z_Im_L_Cyt

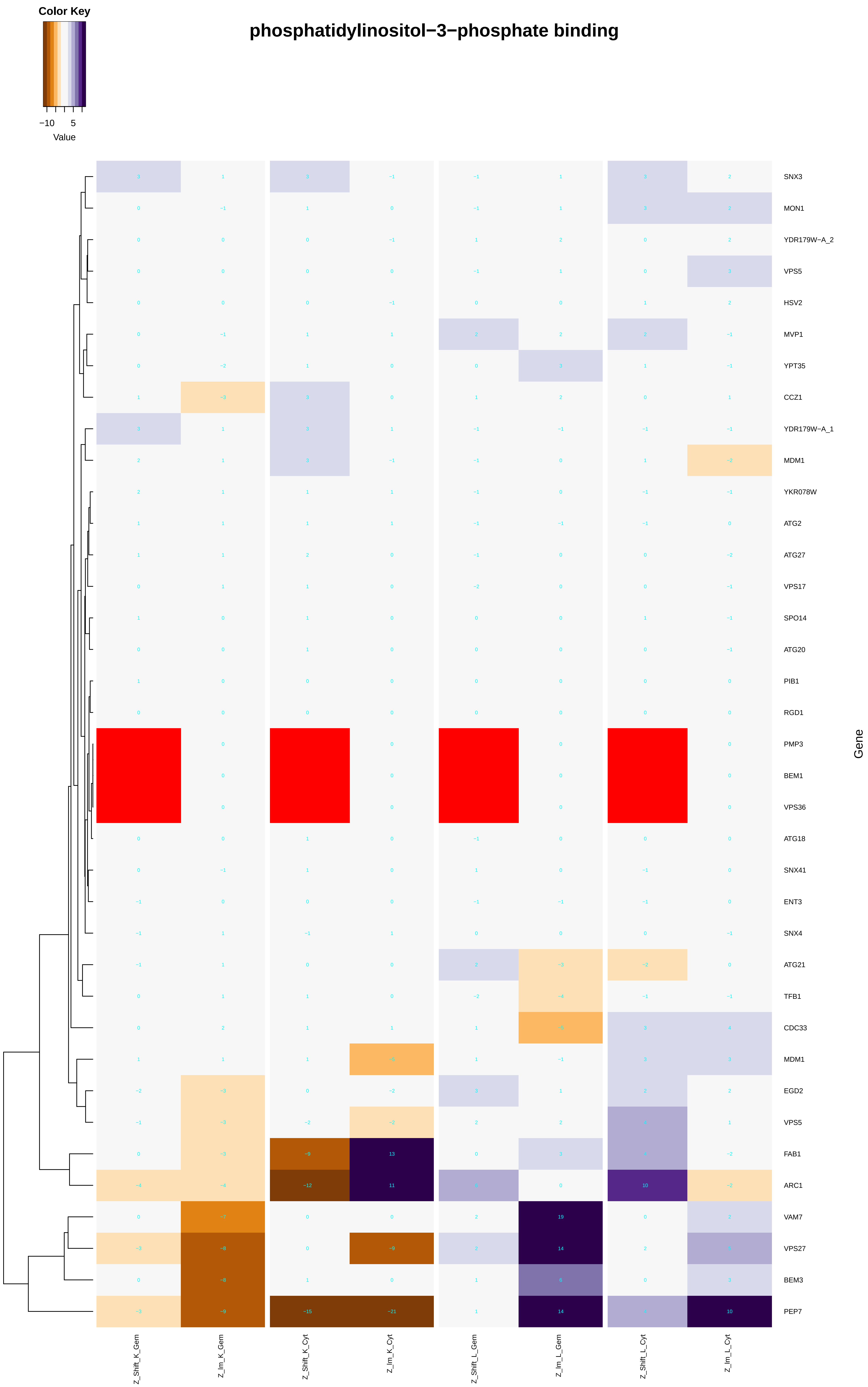
phosphatidylinositol−3−phosphate binding
Color Key
−10
5
Value
SNX3
MON1
YDR179W−A_2
VPS5
HSV2
MVP1
YPT35
CCZ1
YDR179W−A_1
MDM1
YKR078W
ATG2
ATG27
VPS17
SPO14
ATG20
PIB1
RGD1
PMP3
BEM1
VPS36
ATG18
SNX41
ENT3
SNX4
ATG21
TFB1
CDC33
MDM1
EGD2
VPS5
FAB1
ARC1
VAM7
VPS27
BEM3
PEP7
Gene
Z_Shift_K_Gem
Z_Im_K_Gem
Z_Shift_K_Cyt
Z_Im_K_Cyt
Z_Shift_L_Gem
Z_Im_L_Gem
Z_Shift_L_Cyt
Z_Im_L_Cyt

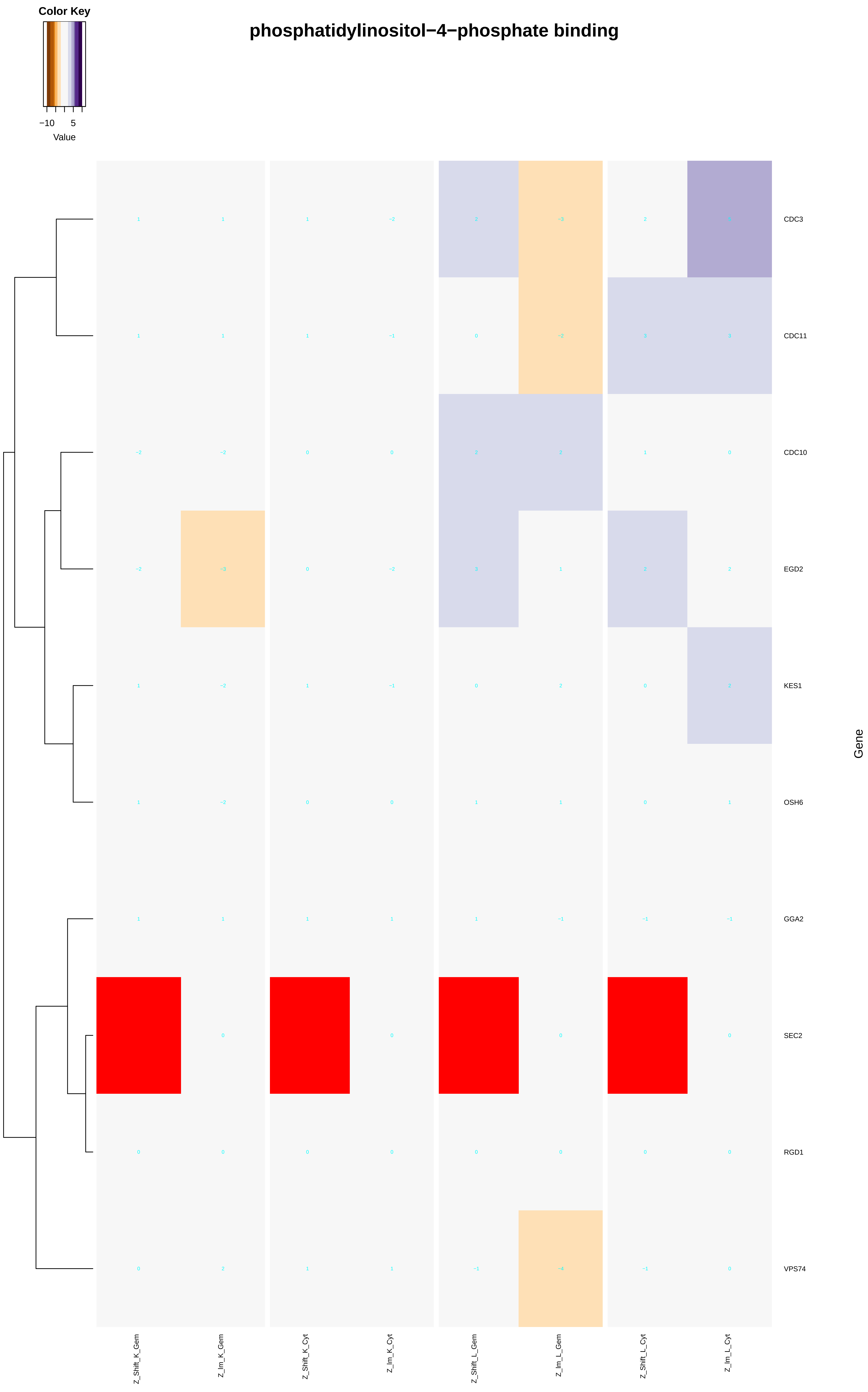
phosphatidylinositol−4−phosphate binding
Color Key
−10
5
Value
CDC3
CDC11
CDC10
EGD2
KES1
OSH6
GGA2
SEC2
RGD1
VPS74
Gene
Z_Shift_K_Gem
Z_Im_K_Gem
Z_Shift_K_Cyt
Z_Im_K_Cyt
Z_Shift_L_Gem
Z_Im_L_Gem
Z_Shift_L_Cyt
Z_Im_L_Cyt

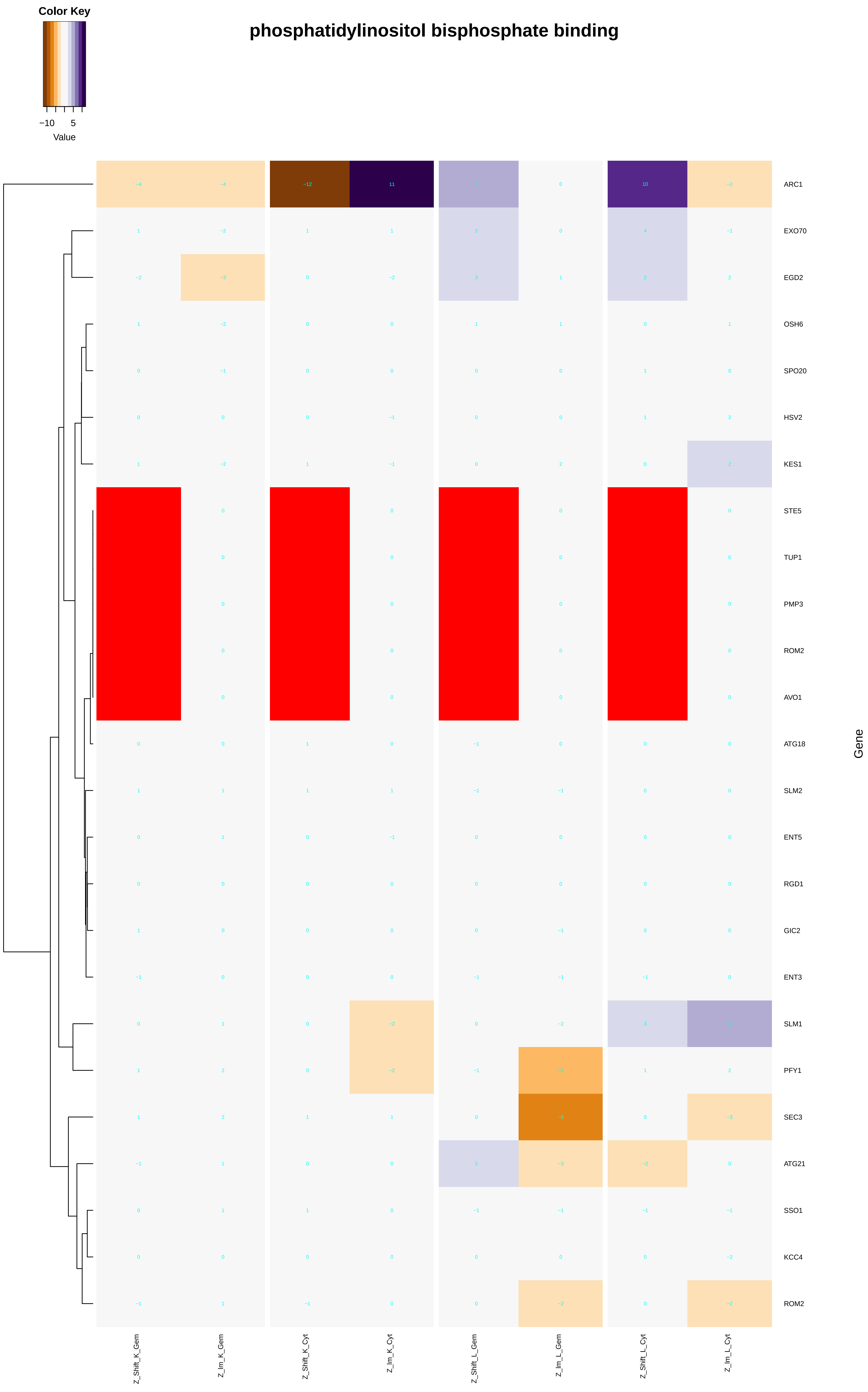

Color Key
−10
5
Value
phosphatidylinositol bisphosphate binding
ARC1
EXO70
EGD2
OSH6
SPO20
HSV2
KES1
STE5
TUP1
PMP3
ROM2
AVO1
ATG18
SLM2
ENT5
RGD1
GIC2
ENT3
SLM1
PFY1
SEC3
ATG21
SSO1
KCC4
ROM2
Gene
Z_Shift_K_Gem
Z_Im_K_Gem
Z_Shift_K_Cyt
Z_Im_K_Cyt
Z_Shift_L_Gem
Z_Im_L_Gem
Z_Shift_L_Cyt
Z_Im_L_Cyt

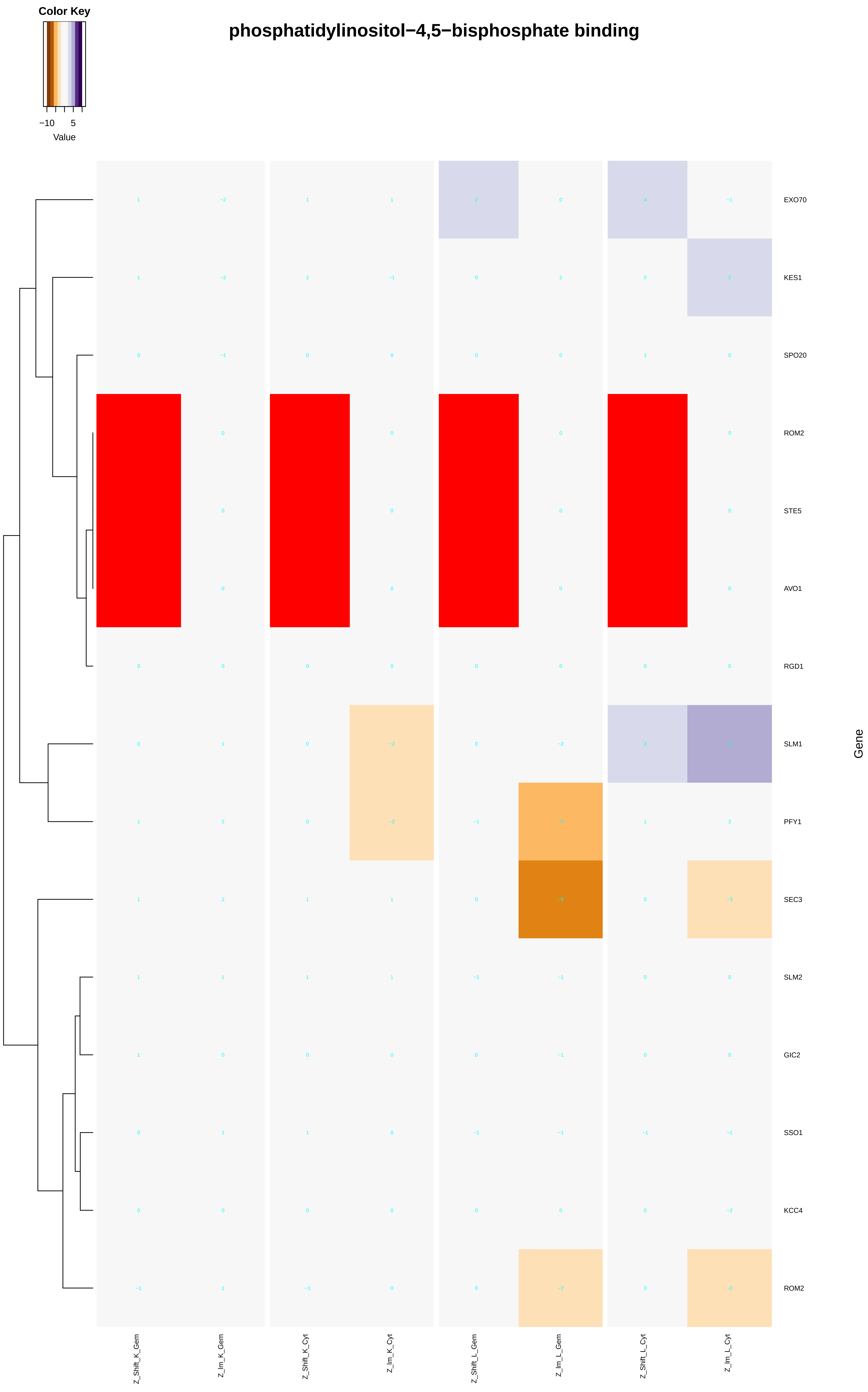
phosphatidylinositol−4,5−bisphosphate binding
Color Key
−10
5
Value
EXO70
KES1
SPO20
ROM2
STE5
AVO1
RGD1
SLM1
PFY1
SEC3
SLM2
GIC2
SSO1
KCC4
ROM2
Gene
Z_Shift_K_Gem
Z_Im_K_Gem
Z_Shift_K_Cyt
Z_Im_K_Cyt
Z_Shift_L_Gem
Z_Im_L_Gem
Z_Shift_L_Cyt
Z_Im_L_Cyt

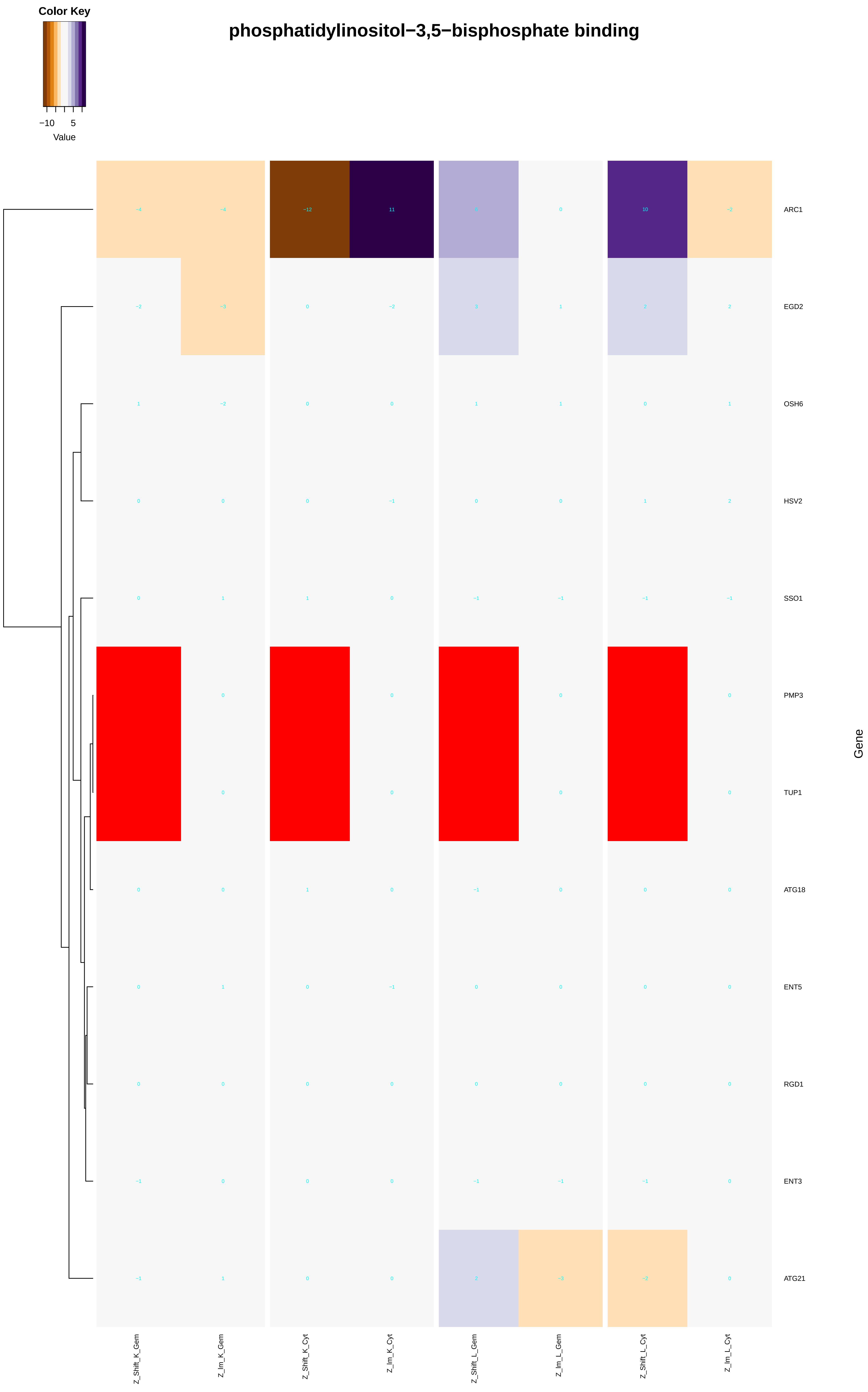

Color Key
−10
5
Value
phosphatidylinositol−3,5−bisphosphate binding
ARC1
EGD2
OSH6
HSV2
SSO1
PMP3
TUP1
ATG18
ENT5
RGD1
ENT3
ATG21
Gene
Z_Shift_K_Gem
Z_Im_K_Gem
Z_Shift_K_Cyt
Z_Im_K_Cyt
Z_Shift_L_Gem
Z_Im_L_Gem
Z_Shift_L_Cyt
Z_Im_L_Cyt
